# Imerslund‐Gräsbeck Syndrome Caused by Compound Heterozygous Mutations in the *AMN* Gene: A Case Report

**DOI:** 10.1002/ccr3.73364

**Published:** 2026-08-19

**Authors:** Cheng Chen, Yijia Min, Xiaoping Ye, Yu Ma, Huiqing Ge, Jingying Chou, Xiaoling Yang, Yingying Cui, Xiaochun Zhang

**Affiliations:** ^1^ Peking University First Hospital Ningxia Women and Children's Hospital (Ningxia Hui Autonomous Region Maternal and Child Health Hospital) Yinchuan Ningxia China; ^2^ Dian Diagnostics Group co., Ltd Shanghai China; ^3^ Peking University First Hospital Beijing China

**Keywords:** *AMN* gene, Imerslund‐Gräsbeck syndrome, megaloblastic anemia, vitamin B_12_

## Abstract

The 7‐year‐old girl had recurrent anemia for 6 years, showing large cell anemia. The parent‐derived AMN double heterozygous mutation was detected to confirm the diagnosis of IGS. The hemogram was normal after intramuscular injection of vitamin B_12_, and there were no other complications.

## 1 Introduction and Background

Imerslund‐Gräsbeck syndrome (IGS), also known as selective vitamin B_12_ (VB_12_) malabsorption with proteinuria and megaloblastic anemia Type I, was first described by Imerslund and Gräsbeck in 1960 [1]. It is a rare autosomal recessive genetic disorder characterized by megaloblastic anemia and neurological damage resulting from impaired VB_12_ absorption, often accompanied by proteinuria, with an incidence of less than 6 cases per 1 million people [2]. The etiology of IGS is primarily linked to mutations in the *AMN* or *CUBN* genes. The *AMN* gene (short for “Amnionless”) is located on human chromosome 14 and encodes a Type I transmembrane protein. This protein forms a complex with cubilin (encoded by the *CUBN* gene), which mediates cellular uptake of VB_12_ [3, 4]. Mutations in *AMN* or *CUBN* disrupt receptor function, thereby impairing VB_12_ absorption. This report describes the clinical characteristics and genetic mechanisms of a child with IGS caused by *AMN* gene CHMs.

## 2 Case History/Examination

**FIGURE 1:**
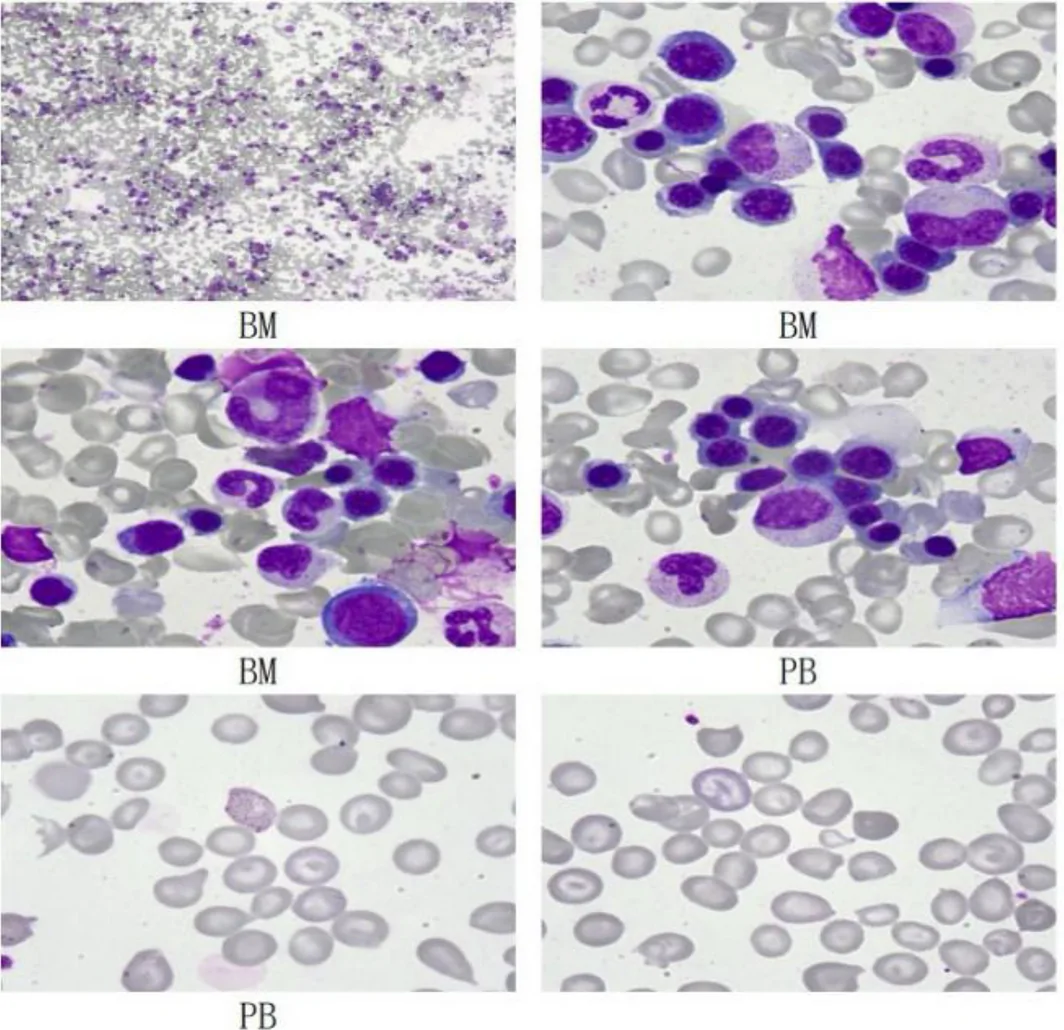
Bone marrow cytology (BM, Bone marrow; PB, Peripheral blood).

The patient was a 7‐year‐old girl who first presented with “anemia” at another hospital 6 years prior. At that time, routine blood tests showed hemoglobin (Hb) 80 g/L and MCV 85 fL, and bone marrow cytology confirmed megaloblastic anemia (Figure 1). After over 2 months of treatment with intramuscular VB_12_ and oral folic acid (FA), her Hb normalized, and medications were discontinued. Subsequent blood work showed generally normal Hb levels, but MCV remained above the normal range.

## 3 Differential Diagnosis, Investigations and Treatment

1. Nutritional anemia: folic acid deficiency, accompanied by complex malnutrition, such as iron deficiency, vitamin B_1_, B_2_, C and protein deficiency, the disease occurs in pregnancy and infancy, vitamin B_12_, folic acid detection decreased, some children may have reduced ferritin; the child has a history of folic acid and vitamin B_12_ deficiency. Regular oral folic acid and vitamin B_12_ hemoglobin can rise to normal, so conside.
2. Malignant anemia: autoimmune destruction of gastric parietal cells, gastric mucosal atrophy leads to lack of internal factors, so that vitamin B_12_ absorption disorders, malignant anemia can be seen in hyperthyroidism, chronic lymphocytic thyroiditis, rheumatoid arthritis, etc.
3. Myelodysplastic syndrome: It is a group of hematopoietic stem cell acquired clonal diseases, often at the same time or successively appear red blood cells, granulocytes and megakaryocytes dysplasia lead to progressive, refractory peripheral blood red blood cells, granulocytes and thrombocytopenia; ineffective hematopoiesis in bone marrow; the main clinical manifestations are anemia, infection or (and) bleeding, which need to be further clarified by bone marrow cytology.
4. Pure red aplastic anemia: a group of rare anemia syndrome that selectively affects the proliferation and differentiation of erythroid progenitor cells in bone marrow, causing simple erythroid hematopoietic failure, and normal myeloid, megakaryocytic, and lymphoid systems. The clinical manifestations are severe progressive anemia, normal red blood cell or mild red blood cell anemia, with significant reduction or absence of reticulocytes, normal or nearly normal peripheral white blood cell and platelet counts.
5. Gene abnormality: a group of anemia caused by deoxyribonucleic acid (DNA) synthesis disorder in large cell anemia, mainly caused by vitamin B_12_ or folic acid deficiency in the body, and also caused by genetic or drug‐acquired DNA synthesis disorder. The patient needs to improve the anemia gene to further clarify.

Four years prior, a school physical exam revealed an Hb of 80 g/L. She received intramuscular VB_12_, oral iron, and FA for more than 3 months. One year prior, routine blood tests showed: Hb 127 g/L, MCV 85 fL, mean corpuscular hemoglobin concentration (MCHC) 28.9 g/L, mean corpuscular hemoglobin (MCH) 340 pg., platelets (PLT) 452 × 10^9^/L, VB_12_ 95.24 pg/mL, and FA 40.74 pg/mL. She was treated with oral FA and VB_12_ for 4 months.

Four days before admission, she developed vomiting, loose stools, and abdominal pain. On admission, physical examination showed a blood pressure of 99/62 mmHg, no skin rash or petechiae, no palpable superficial lymphadenopathy, and pale lips, conjunctiva, and nail beds. Cardiopulmonary, abdominal, and neurological exams were normal. Routine blood tests on admission showed: white blood cells (WBC) 4.50 × 10^9^/L, neutrophils 31.5%, Hb 85 g/L, MCV 103.00 fL, PLT 204 × 10^9^/L; FA 8.83 ng/mL, VB_12_ 626.71 pg/mL, ferritin 182.16 ng/mL; blood biochemistry showed lactate dehydrogenase 1051 U/L. Abdominal ultrasound and chest X‐ray were normal.

**FIGURE 2:**
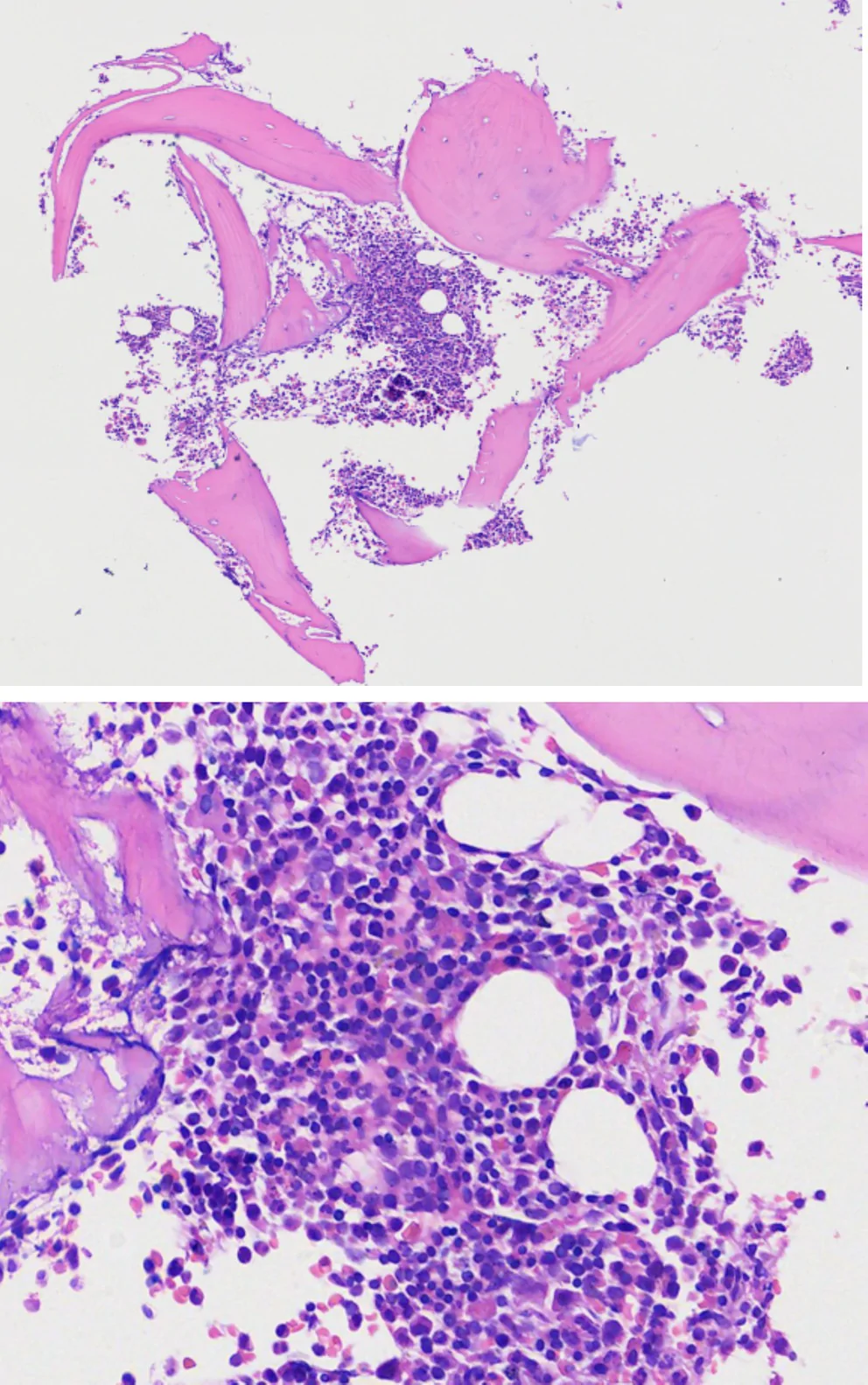
Findings from bone marrow biopsy.

Bone marrow cytology revealed megaloblastoid changes in neutrophilic metamyelocytes and band neutrophils, active erythroid hyperplasia, occasional megaloblasts, and visible macro‐ovalocytes. Bone marrow biopsy (Figure 2) showed generally normal marrow hyperplasia, with hematopoietic components accounting for ~90% and fat ~10%; a decreased myeloid‐erythroid ratio; granulocytic lineage predominantly consisting of mid‐late immature and subsequent stage cells, with no obvious abnormal localization of immature precursor cells; erythroid lineage mainly composed of mid‐late immature cells, with erythroid hematopoietic islands present; a normal megakaryocyte count (~3/HPF), predominantly segmented‐nucleus cells; and scattered lymphocytes and plasma cells.

**FIGURE 3:**
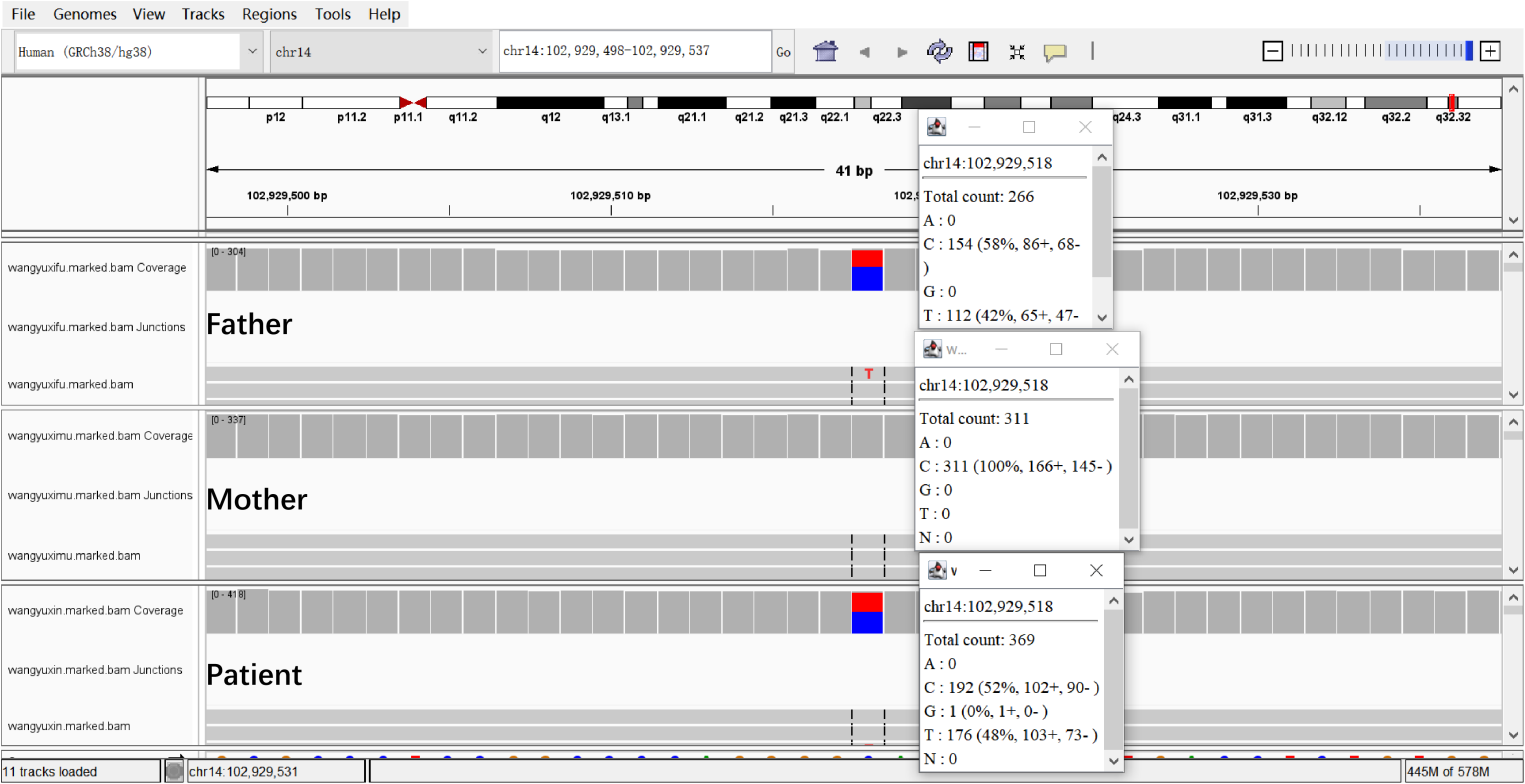
Tested loci in the patient and parents (c.742C>T) Bam map.

**FIGURE 4:**
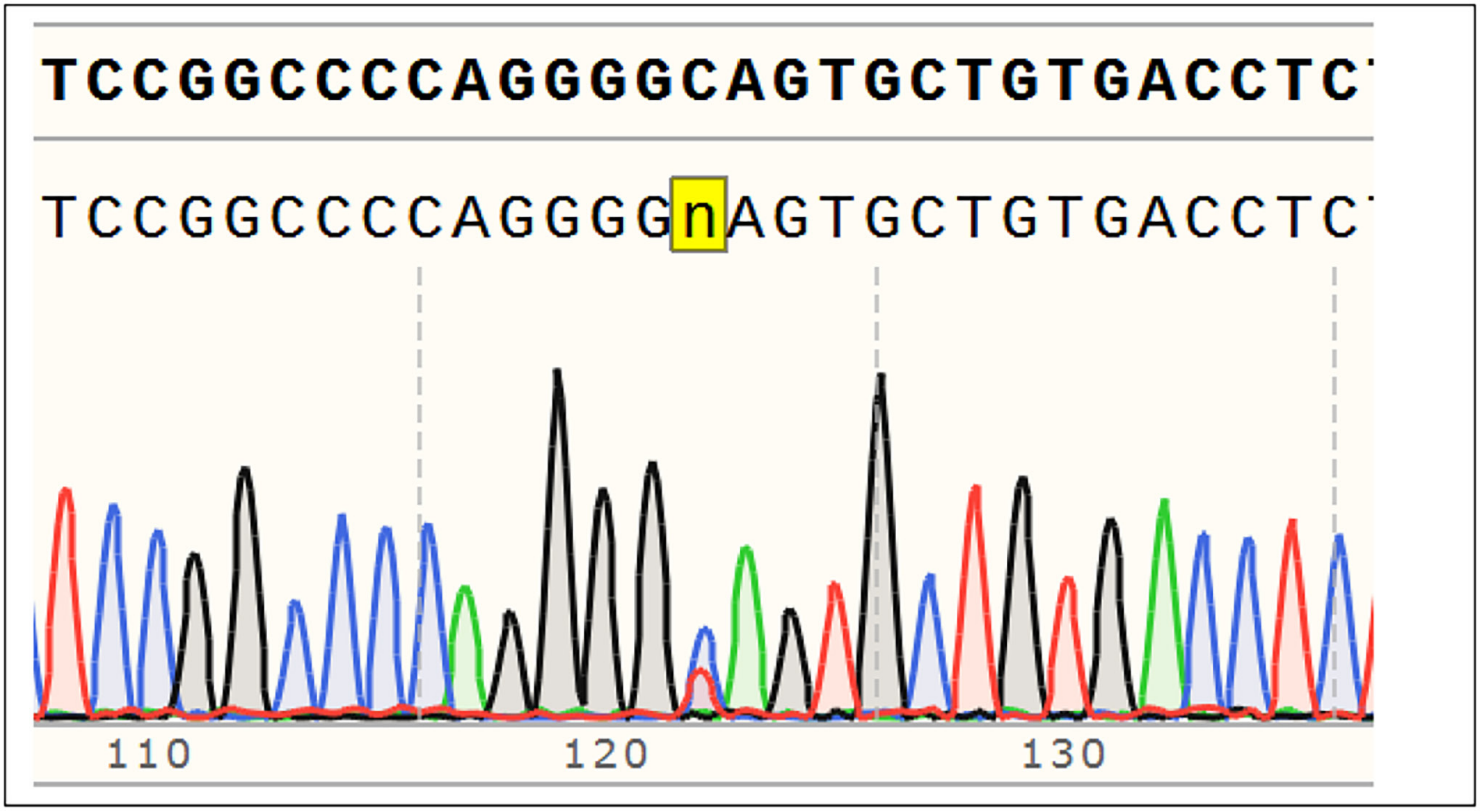
Sanger sequencing map of the patient's tested locus (heterozygous mutation c.742C>T).

**FIGURE 5:**
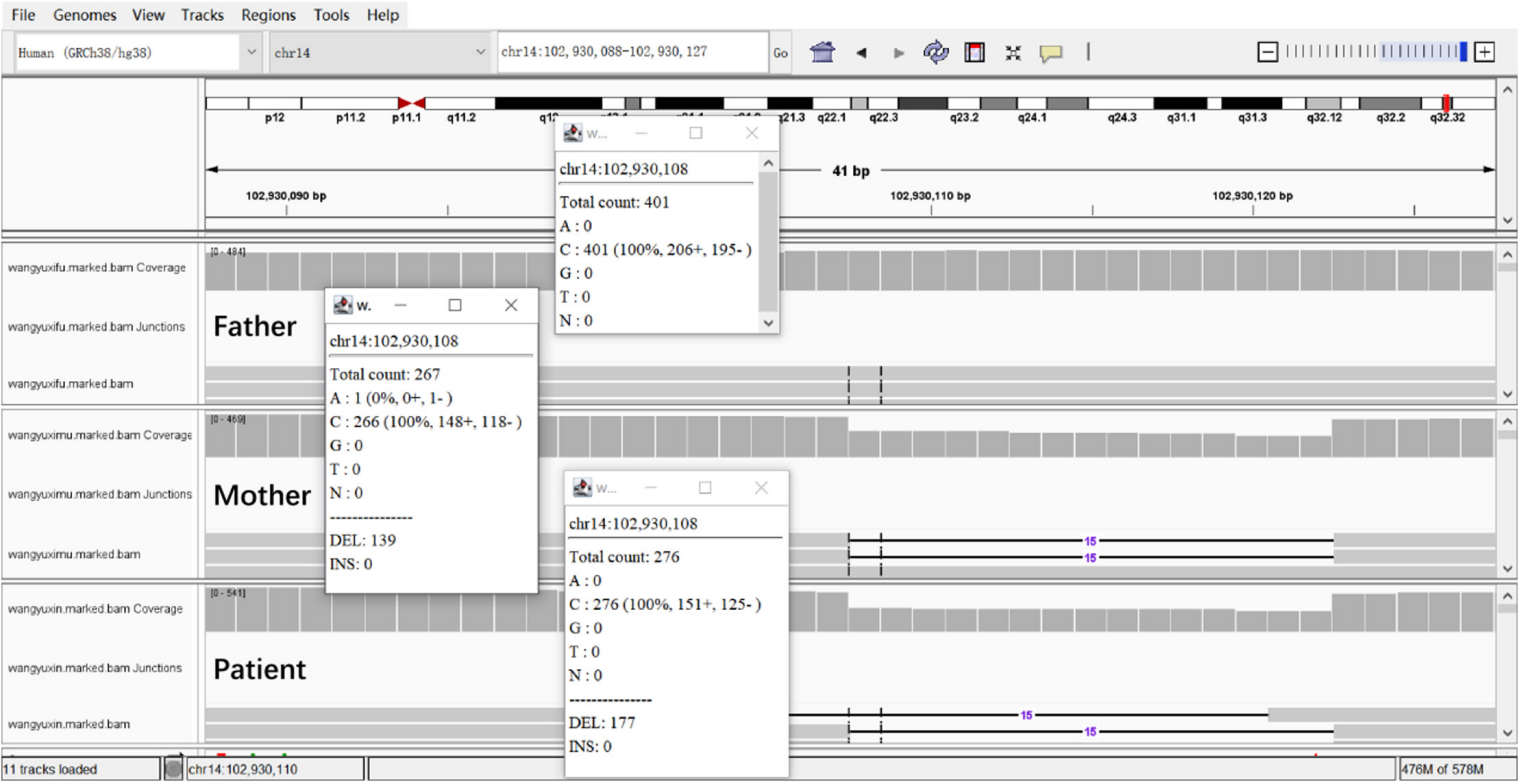
Tested loci in the patient and parents (c.1006 + 34_1007‐31del) Bam map.

**FIGURE 6:**
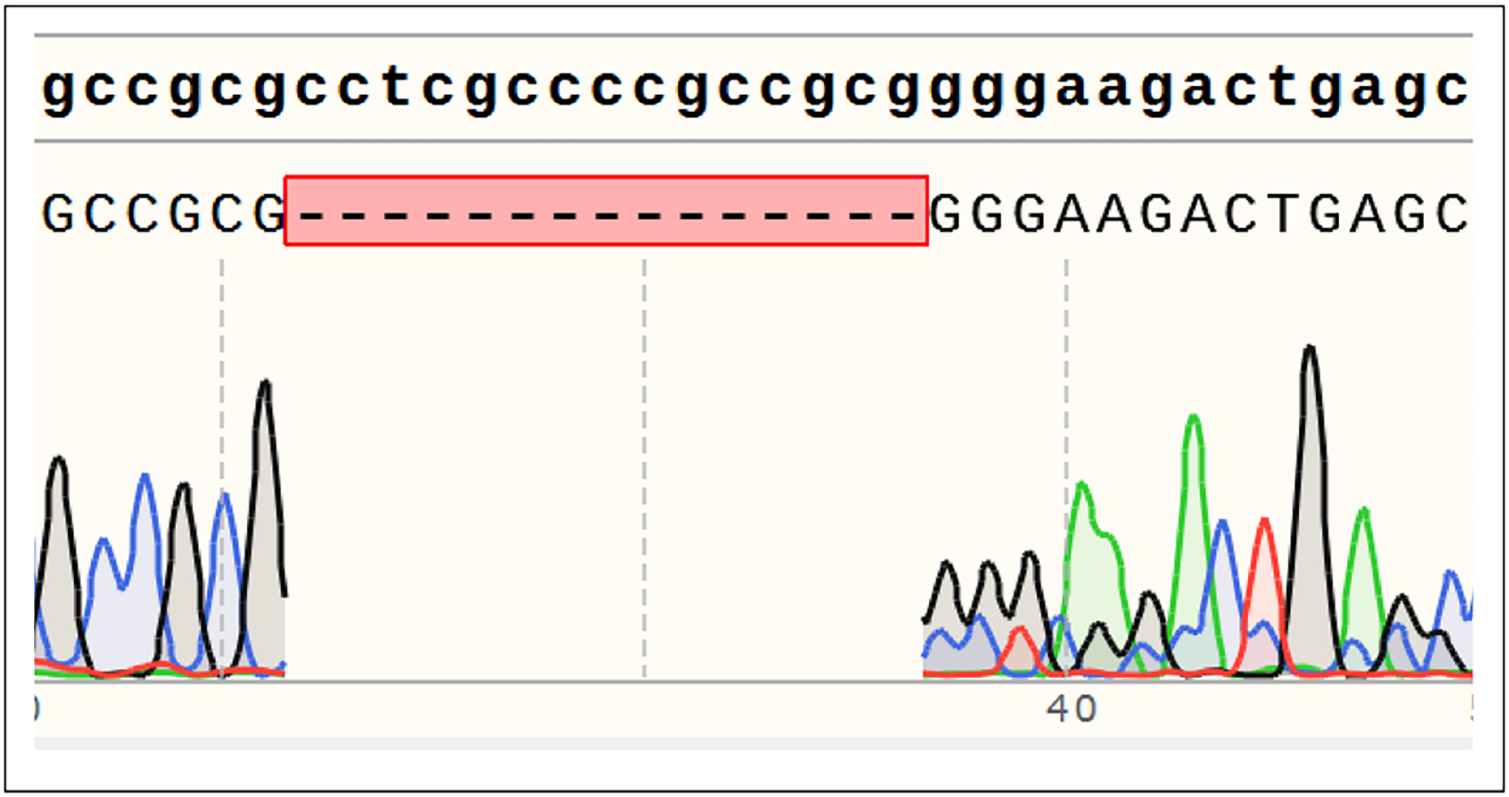
Sanger sequencing map of the patient's tested locus (heterozygous mutation c.1006 + 34_1007‐31del).

With parental informed consent and approval from the hospital ethics committee, 3 mL of peripheral venous blood was collected from the patient and her parents for hematological genetic testing and Sanger sequencing verification at Dian Diagnostics Group Co. Ltd. Results identified CHMs in the *AMN* gene on chromosome 14: c.742C>T (p.Q248X) and c.1006 + 34_1007‐31del (Figures 3, 4, 5, 6).

**FIGURE 7:**
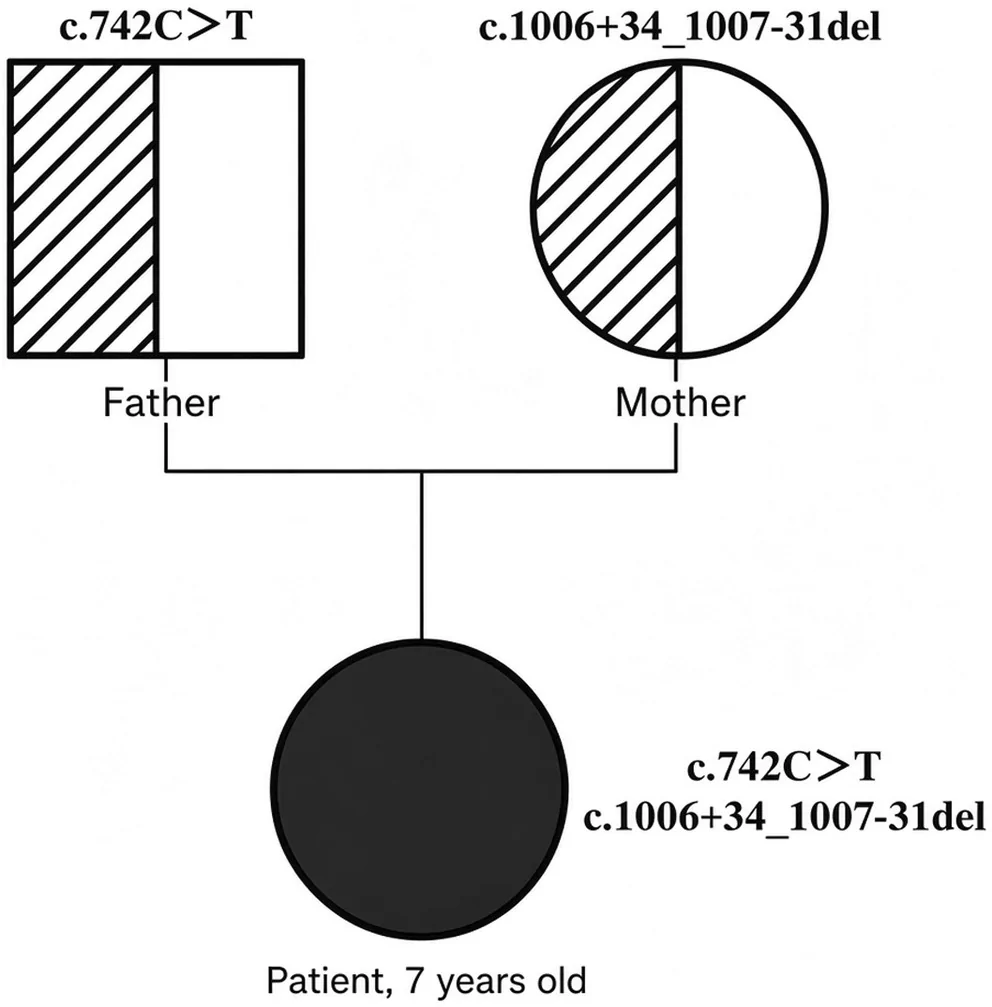
Family pedigree diagram.

The heterozygous nonsense mutation c.742C>T (p.Q248X) in exon 7 (NM_030943.3, exon 7) introduces a premature stop codon at amino acid 248, resulting in a truncated protein, and was inherited from the father. The c.1006 + 34_1007‐31del mutation (a 15 bp deletion in intron 9) is a frameshift mutation affecting gene splicing, causing exon 9 skipping and a premature termination codon, inherited from the mother. Both mutations are associated with IGS in HGMDPro, predicted to be deleterious by MutationTaster, and classified as pathogenic variants according to ACMG guidelines and ClinVar pathogenicity assessments.

## 4 Outcome and Follow‐Up

The child was diagnosed with megaloblastic anemia, with the etiology identified as compound heterozygous mutations in the *AMN* gene: a heterozygous mutation of c.742C>T (p.Q248X) and a heterozygous mutation of c.1006 + 34_1007‐31del (Figure 7). After confirming the cause, the child was administered monthly intramuscular injections of 1 mg vitamin B_12_ for treatment. Currently, she remains under continuous treatment and follow‐up; the hemoglobin level has been maintained within the normal range, and no abnormalities or complications in other systems have been detected so far.

## 5 Discussion

IGS is a rare autosomal recessive disorder caused by mutations in *AMN* or *CUBN* genes, leading to selective VB_12_ malabsorption and proteinuria [5]. With a prevalence of < 6/1,000,000, it primarily affects populations in the Mediterranean region [6]. Typical clinical features include megaloblastic anemia responsive to parenteral VB_12_, selective low‐molecular‐weight proteinuria (present in ~50% of patients), growth retardation, and recurrent infections, with an average age of onset of 3.5 years [7].

In this case, the child presented with recurrent anemia responsive to intramuscular VB_12_. WES identified *AMN* gene CHMs: c.742C>T (p.Q248X) and c.1006 + 34_1007‐31del, consistent with the autosomal recessive inheritance pattern of IGS. While each mutation has been reported individually in the literature [8, 9]—with both presenting as recurrent anemia responsive to VB_12_ treatment—this is the first report of their combination as CHMs. This case has expanded the genotype spectrum of AMN‐related IGS and the database of genotype–phenotype associations.

VB_12_ (cobalamin) acts as a coenzyme for methionine synthase and L‐methylmalonyl‐CoA mutase. Deficiency leads to homocysteinemia and methylmalonic acidemia, causing megaloblastic anemia, neurological damage, and metabolic disorders [9]. In children, VB_12_ deficiency manifests as growth retardation or regression, hypotonia, and lethargy. Gene mutations disrupt the receptor function for the intestinal factor‐vitamin B_12_ complex. The amnionless subunit (encoded by *AMN* on 14q32) and cubilin subunit (encoded by *CUBN* on 10p12.1) form a receptor complex involved in VB_12_ transmembrane transport and renal tubular protein reabsorption [10].

As of 2025, the dbSNP database has recorded 72 pathogenic variants in *CUBN* and 38 in *AMN*, reflecting genotypic diversity. Previous studies have shown that c.742C > T (p.Q248X) is a common pathogenic variant in Chinese children with IGS, and there is significant phenotypic heterogeneity among patient [11]. Liu et al. first reported a Chinese boy carrying a homozygous c.742C > T variant. His clinical manifestations included growth retardation, severe macrocytic anemia, and persistent proteinuria [12]. Parenteral administration of cobalamin was effective in treatment, but tubular proteinuria persisted during long‐term follow‐up [12]. Qin et al. further reported two sibling patients from a consanguineous family carrying a homozygous c.742C > T variant, and their clinical manifestations were significantly different [13]. The elder sister presented with severe pancytopenia, hemolysis, recurrent respiratory infections, growth retardation, and persistent proteinuria; the younger sister, who also had α‐thalassemia, only showed simple anemia without obvious proteinuria [13]. When this variant combines with different trans‐alleles, it can lead to varying degrees of neurological sequelae, suggesting the roles of allelic interaction and genetic modifiers. The intron deletion variant c.1006 + 34_1007‐31del has been reported in multiple pediatric cases, and there are great individual differences in the age of onset and the severity of clinical conditions. Children carrying this variant may present with severe pancytopenia, or develop simple early proteinuria before the appearance of anemia and mild neurological symptoms [5, 14]. Since deep intronic structural variants such as c.1006 + 34_1007‐31del are often missed by routine NGS panels that only cover exons, for patients with a high clinical suspicion of IGS but only one pathogenic coding‐region variant detected, it is recommended to perform full‐intron sequencing or copy number variant detection. The patient in this case, similar to carriers of any of the aforementioned variants reported previously, presents with the typical core clinical manifestations of Imerslund‐Gräsbeck syndrome (IGS): recurrent macrocytic anemia, typical megaloblastic changes in the erythroid and granulocytic lineages in the bone marrow, elevated lactate dehydrogenase (LDH) due to ineffective hematopoiesis, and no obvious neurological damage. Consistent with previous research findings, oral folic acid or oral cobalamin supplementation only leads to transient hematological remission, causing the disease to relapse repeatedly. The patient in this case also exhibits significant phenotypic heterogeneity. First, almost all IGS patients carrying the c.742C>T or c.1006 + 34_1007‐31del variants have characteristic renal manifestations—persistent tubular proteinuria, but the proband in this case shows no such manifestations at all, suggesting that even with two AMN loss‐of‐function alleles, renal‐related manifestations may still show incomplete penetrance. Second, the patient presented with repeated vomiting as the initial symptom of acute exacerbation, which is rarely reported as the initial manifestation in IGS‐related literature. This unique clinical phenotype expands the genotype–phenotype spectrum of double AMN loss‐of‐function variants, indicating that there can still be significant clinical heterogeneity among patients even with alleles of known pathogenicity. The biallelic pathogenic combination of c.742C > T and c.1006 + 34_1007‐31del in this case meets the ACMG criteria for pathogenic variants (both alleles meet PVS1; the low population allele frequency meets PM2; the evidence of trans‐configuration meets PM3; the intron deletion variant is supported by splicing bioinformatics prediction).

With the adoption of next‐generation sequencing (NGS), the challenge of differentiating IGS from intrinsic factor‐deficient megaloblastic anemia has gradually been addressed. The application of NGS technology has significantly improved the diagnostic accuracy of rare genetic diseases, guiding genetic counseling and preventing metabolic crises or irreversible nerve damage caused by misdiagnosis, making the treatment more precise.

Lifelong monthly intramuscular VB_12_ injections are the standard treatment for IGS, and supra‐physiological oral doses may be partially effective via alternative absorption pathways [15]. Early intervention can reverse anemia and neurological symptoms, though some patients may experience persistent proteinuria or mild hemolysis post‐treatment [16]. Most patients have a favorable long‐term prognosis, but rare complications such as thrombotic thrombocytopenic purpura‐like manifestations or pancytopenia have been reported.

In conclusion, IGS diagnosis is based on VB_12_ levels, and genetic testing should be conducted promptly. When conventional treatment methods for childhood anemia yield poor results, the possibility of hereditary anemia should be considered, and genetic testing is helpful for the definitive diagnosis of rare hereditary metabolic diseases.

## Author Contributions

**Xiaoping Ye:** methodology. **Yijia Min:** methodology. **Yu Ma:** project administration, data curation, investigation. **Cheng Chen:** conceptualization, visualization, data curation, writing – original draft, project administration, methodology. **Yingying Cui:** project administration, data curation, visualization, investigation. **Huiqing Ge:** project administration, data curation, investigation. **Jingying Chou:** project administration, data curation, investigation. **Xiaochun Zhang:** writing – review and editing, project administration, data curation, validation, visualization, investigation, methodology, conceptualization. **Xiaoling Yang:** project administration, data curation, writing – review and editing.

## Funding

The authors have nothing to report.

## Ethics Statement

The authors have nothing to report.

## Consent

Written informed consent was obtained from the patient's legal guardian for publication of this case report and any accompanying images. A copy of the written consent is available for review by the Editor‐in‐Chief of this journal.

## Conflicts of Interest

The authors declare no conflicts of interest.

## Data Availability

The data that support the findings of this study are available from the corresponding author upon reasonable request.
